# Venous thromboembolism chemical prophylaxis after skull base surgery

**DOI:** 10.1007/s00701-024-06035-9

**Published:** 2024-04-03

**Authors:** Mueez Waqar, Omar Yaseen, Annabel Chadwick, Jing Xian Lee, Ghazn Khan, D. Gareth Evans, Daniel Horner, Archana Jaiswal, Simon Freeman, Rajiv Bhalla, Simon Lloyd, Charlotte Hammerbeck-Ward, Scott A. Rutherford, Andrew T. King, Omar N. Pathmanaban

**Affiliations:** 1 Department of Neurosurgery, Manchester Centre for Clinical Neurosciences, Northern Care Alliance NHS Foundation Trust, Salford, UK; 2 Geoffrey Jefferson Brain Research Centre, Division of Neuroscience, School of Biological Sciences, Faculty of Biology, Medicine and Health, The University of Manchester, Manchester, UK; 3 Department of Neurogenetics, Manchester Centre for Genomic Medicine, St Mary’s Hospital, Central Manchester University Hospitals NHS Foundation Trust, Manchester, UK; 4 Department of Neurocritical Care, Manchester Centre for Clinical Neurosciences, Northern Care Alliance NHS Foundation Trust, Salford, UK; 5 Department of Otorhinolaryngology, Manchester Centre for Clinical Neurosciences, Northern Care Alliance NHS Foundation Trust, Salford, UK

**Keywords:** Venous thromboembolism, Skull base, Deep vein thrombosis, Pulmonary embolism, Cranial base, Vestibular schwannoma, Neurosurgery, Otolaryngology, Hematoma

## Abstract

**Purpose:**

There is no guidance surrounding postoperative venous thromboembolism (VTE) prophylaxis using pharmacological agents (chemoprophylaxis) in patients undergoing skull base surgery. The aim of this study was to compare VTE and intracranial haematoma rates after skull base surgery in patients treated with/without chemoprophylaxis.

**Methods:**

Review of prospective quaternary centre database including adults undergoing first-time skull base surgery (2009–2020). VTE was defined as deep vein thrombosis (DVT) and pulmonary embolism (PE) within 6 months of surgery. Multivariate logistic regression was used to determine factors predictive of postoperative intracranial haematoma/VTE. Propensity score matching (PSM) was used in group comparisons.

**Results:**

One thousand five hundred fifty-one patients were included with a median age of 52 years (range 16–89 years) and female predominance (62%). Postoperative chemoprophylaxis was used in 81% of patients at a median of 1 day postoperatively. There were 12 VTE events (1.2%), and the use of chemoprophylaxis did not negate the risk of VTE entirely (*p* > 0.99) and was highest on/after postoperative day 6 (9/12 VTE events). There were 18 intracranial haematomas (0.8%), and after PSM, chemoprophylaxis did not significantly increase the risk of an intracranial haematoma (*p* > 0.99). Patients administered chemoprophylaxis from postoperative days 1 and 2 had similar rates of intracranial haematomas (*p* = 0.60) and VTE (*p* = 0.60), affirmed in PSM.

**Conclusion:**

Postoperative chemoprophylaxis represents a relatively safe strategy in patients undergoing skull base surgery. We advocate a personalised approach to chemoprophylaxis and recommend it on postoperative days 1 or 2 when indicated.

**Supplementary Information:**

The online version contains supplementary material available at 10.1007/s00701-024-06035-9.

## Introduction

Venous thromboembolism (VTE) is the composite of deep vein thrombosis (DVT) and pulmonary embolism (PE), which are serious and potentially fatal postoperative complications. Such complications are potentially preventable. The risk of VTE can be reduced through mechanical thromboprophylaxis with compression stockings and intermittent pneumatic compression, along with pharmacological thromboprophylaxis (chemoprophylaxis). These measures are often used in conjunction as there is limited evidence for mechanical thromboprophylaxis alone [6, 21]. In contrast, there is strong evidence of benefit for chemoprophylaxis in hospitalised patients [19], though data for patients undergoing intracranial surgery are limited.

The American Heart Association recommends against using chemoprophylaxis in patients undergoing major neurosurgical procedures, except in those deemed at high risk, such as those experiencing prolonged immobility [5]. These guidelines are open to interpretation and do not specifically address skull base surgery, which is also the case for European guidelines [3]. In the UK, the National Institute for Health and Care Excellence (NICE) guidelines suggest initiation of chemoprophylaxis 24–48 h after cranial surgery where benefits outweigh risks [20]. As such, decision-making surrounding chemoprophylaxis must be made on a case-by-case basis by balancing the benefits of VTE reduction with the potentially increased risk of bleeding events. Meta-analyses and prior institutional series including neurosurgical patients from different subspecialties have demonstrated that pharmacological thromboprophylaxis with heparin agents can be used postoperatively to decrease the rate of VTE with minimal risk of bleeding [4, 10, 11, 14, 16, 23]. However, there is sparse outcome data for patients undergoing skull base surgery. In this cohort of patients, the potential risks of chemoprophylaxis include postoperative haematoma with serious associated morbidity due to the proximity of the brainstem, optic nerves and critical neurovascular structures. This must be balanced against the VTE risk inferred by the long operative times in this patient group, reported as an independent risk factor for VTE in population-based studies [17]. Indeed, the VTE risk in skull base patients can be as high as 10% [2].

We recently presented data on the benefits of early chemoprophylaxis in patients undergoing trans-sphenoidal pituitary surgery [25]. Patients undergoing skull base surgery represent a distinct cohort from these patients, given that their operative time is significantly longer, with greater intra-arachnoid brain and vessel manipulation. It is therefore important to present data in this specific patient group. Our institution represents a quaternary and national referral centre for skull base pathology in the United Kingdom. The aims of this study were to describe our experience of chemoprophylaxis following skull base surgery, to describe the absolute risks of haematoma/VTE events in patients treated with/without chemoprophylaxis and to provide guidance and recommendations from our experience.

## Methods

Institutional review board approval was obtained. Patient consent was not required for this study type. We interrogated a prospectively maintained electronic database of locally performed skull base operations. All adults (age ≥ 16 years) undergoing first-time skull base surgery between April 2009 and December 2020 were included. Patients with redo operations were excluded. Patients undergoing trans-sphenoidal pituitary operations were also excluded from this study and analysed separately [25].

Electronic patient records were reviewed and data extracted on demographics, histological diagnosis, operative intervention, postoperative course (including use of chemoprophylaxis) and the development of VTE or postoperative haematomas. We defined VTE as that occurring within 6 months of surgery. This encompassed DVT and PE. The time limitation to our definition of VTE excluded only one upper limb deep vein thrombosis that occurred two years postoperatively from total VTE events. In accordance with national standards and the National Health Service contract, the institution thrombosis committee maintains a separate electronic and prospectively maintained database of VTE-positive events occurring during hospital admission or after hospital discharge, following inpatient admission (Hospital acquired thrombosis). This dataset was cross-referenced to identify any missed VTE events within the included cohort.

At our institution, antiplatelet medication is discontinued 1 week prior to surgery. Anticoagulant medication is discontinued 5 days (warfarin) or 48 h (novel oral anticoagulants) before surgery. Postoperatively, patients are nursed in a specialist monitored bed for at least 24 h. Patients are encouraged to mobilise postoperatively, and there are no bed rest restrictions. The decision to commence postoperative chemoprophylaxis is made after the procedure on daily patient rounds. Potential factors that can influence this decision and the exact day of commencement (e.g. postoperative day 1 vs. subsequent days vs. not at all) include the degree of difficulty encountered with intraoperative haemostasis, coagulation state, preoperative comorbidities and antiplatelet/anticoagulant use and patient mobility.

The preferred low molecular weight heparin (LMWH) agent at our institute is tinzaparin, with dose adjustment to weight and renal function, administered subcutaneously once daily at 1800 h. Chemoprophylaxis is used together with mechanical prophylaxis at our institute; all patients had mechanical prophylaxis with Flowtron® boots (intraoperatively) and TED stockings from hospital admission to discharge as per our institutional protocol. Chemoprophylaxis was discontinued on the day of discharge, with no routine discharge course.

Imaging is not routinely performed in the immediate postoperative period unless clinically indicated. Therefore, postoperative haematomas described in the present study were clinically detected (e.g. with a deterioration in neurology for intracranial haematomas) and confirmed with subsequent imaging where relevant.

Statistical analysis was performed in R version 4.0.5 (R Foundation for Statistical Computing; Vienna, Austria). Descriptive statistics were used to describe patient cohort characteristics. Categorical variables were compared using tests of proportions (Fisher’s exact and Chi-squared). Forward-stepwise multivariate logistic regression was performed to evaluate factors predictive of postoperative intracranial/any haematoma formation. Propensity score matching was used to compare patient groups (chemoprophylaxis versus no chemoprophylaxis and chemoprophylaxis on postoperative day 1 versus day 2) to create 1:1 matching cohorts based age, gender, prior antiplatelet/anticoagulant use, procedure type and surgical approach.

## Results

One thousand five hundred fifty-one patients were included. Patient characteristics are shown in Table 1. The median age was 52 years (range 16–89 years). There was a female predominance (*N* = 955, 62%). Antiplatelets and anticoagulants were used by 7% of patients overall. The most common skull base surgical approaches included retrosigmoid (*N* = 538, 35%) and translabyrinthine/transmastoid (*N* = 514, 33%). The majority of procedures were performed for oncological lesions (*N* = 926, 60%) or microvascular decompression (*N* = 370, 24%).

**Table 1:** Patient characteristics across 1551 de novo skull base cases. *This category includes multiple tumour types including neuroendocrine tumours, neuroblastomas, paragangliomas, nerve sheath tumours, craniopharyngiomas, gliomas, osteoblastomas, juvenile angiofibromas, melanocytomas and haemangiomas

Age (years)	
Median	52
Range	16–89
Gender	
Male	596 (38%)
Female	955 (62%)
Antiplatelets and anticoagulants	
Total	112 (7%)
Aspirin	75 (5%)
Clopidogrel	9 (< 1%)
Warfarin	22 (1%)
Preoperative thrombocytopenia (< 150/microlitre)	40 (3%)
Surgical approach	
Translabyrinthine/transmastoid	514 (33%)
Retrosigmoid	538 (35%)
Foramen magnum decompression	177 (11%)
Frontal/pterional	149 (10%)
Endonasal	75 (5%)
Other	98 (6%)
Type of procedure/histology	
Oncology	927 (60%)
Vestibular Schwannoma	482 (31%)
Meningioma	310 (20%)
Dermoid/epidermoid	27 (2%)
Chordoma	18 (1%)
Cholesteatoma	18 (1%)
Other*	72 (5%)
Microvascular decompression	370 (24%)
Cyst and CSF-related	254 (16%)
Foramen magnum decompression	177 (11%)
Cyst and CSF leak repair	77 (5%)
Chemical VTE prophylaxis	
Yes	1249 (81%)
No	302 (19%)

Postoperative chemoprophylaxis was used in the majority of patients (*N* = 1249, 81%) at a median of 1 day postoperatively (range 1–16 days). Indeed, 81% of cases (1014/1249) had chemoprophylaxis commenced on the first postoperative day. Chemoprophylaxis was employed with tinzaparin (1217, 97%; median dose 4500 units once daily) or enoxaparin (32, 3%; median dose 40 mg once daily). The median duration of chemoprophylaxis was 4 days (range 1–217 days), and chemoprophylaxis was routinely discontinued at discharge.

Within the study period, there was a change in practice from infrequent use of postoperative chemoprophylaxis (2009–2011, chemoprophylaxis used in 157/360 patients, 44%) to more routine use (2018–2020, chemoprophylaxis used in 312/329 patients, 95%).

Table 2 shows differences between patients who did/did not receive chemoprophylaxis. The chemoprophylaxis group had significantly more oncology cases (63% vs. 44%), whereas the no chemoprophylaxis group had a relative excess of foramen magnum decompressions (10% vs. 16%; Chi-squared, *χ*2 = 38.3, *p* < 0.001). There were also more frontal/pterional approaches in the chemoprophylaxis group (11% vs. 4%; Chi-squared, *χ*2 = 26.1, *p* < 0.001).

**Table 2:** Comparison between patients that did/did not receive chemoprophylaxis. The chemoprophylaxis group had significantly more oncology cases, fewer foramen magnum decompressions and more frontal/pterional approaches. This table groups 11 patients (*) that developed haematom as prior to commencement of chemoprophylaxis in the chemoprophylaxis group. There was no significant difference between these groups in terms of postoperative haematoma formation

	Chemoprophylaxis (*N* = 1249)	No chemoprophylaxis (*N* = 302)	Comparison
Age (years)			
Median	51	53	*t*-test, *t* = 0.22, *p* = 0.83
Mean	51	51	
Range	16–88	16–89	
Gender			
Male	474 (38%)	122 (40%)	Fisher’s exact, *p* = 0.43
Female	775 (62%)	180 (60%)	
Pre-op antiplatelets or anticoagulants			
No	1151 (92%)	288 (95%)	Fisher’s exact, *p* = 0.06
Yes	98 (8%)	14 (5%)	
Thrombocytopenia			
No	1217 (97%)	294 (97%)	Fisher’s exact, *p* = 0.84
Yes	32 (3%)	8 (3%)	
Surgical approach			
Translabyrinthine/transmastoid	417 (33%)	97 (32%)	**Chi-squared, *χ*2 = 26.1, *p* < 0.001**
Retrosigmoid	425 (34%)	113 (37%)	
Foramen magnum decompression	128 (10%)	49 (16%)	
Frontal/pterional	138 (11%)	11 (4%)	
Endonasal	66 (5%)	9 (3%)	
Other	75 (6%)	23 (8%)	
Type of procedure/histology			
Oncology	794 (63%)	133 (44%)	**Chi-squared, *χ*2 = 38.3, *p* < 0.001**
Vestibular Schwannoma	407 (33%)	75 (25%)	
Meningioma	286 (23%)	24 (8%)	
Dermoid/epidermoid	21 (2%)	6 (2%)	
Chordoma	18 (1%)	0 (0%)	
Cholesteatoma	10 (1%)	8 (3%)	
Other	52 (4%)	20 (7%)	
Microvascular decompression	271 (22%)	99 (33%)	
Cyst and CSF-related	184 (15%)	70 (23%)	
Foramen magnum decompression	128 (10%)	49 (16%)	
Cyst and CSF leak repair	56 (5%)	21 (7%)	
Haematoma*			
Superficial extracranial	3 (< 1%)	1 (< 1%)	Fisher’s exact, *p* = 0.49
Intracranial	15 (1%)	3 (1%)	
Abdominal	9 (< 1%)	0 (0%)	
Venous thromboembolism			
Deep vein thrombosis	3 (< 1%)	0 (0%)	Fisher’s exact, *p* > 0.99
Pulmonary embolism	7 (< 1%)	2 (1%)	

### Postoperative haematomas

**Fig. 1:**
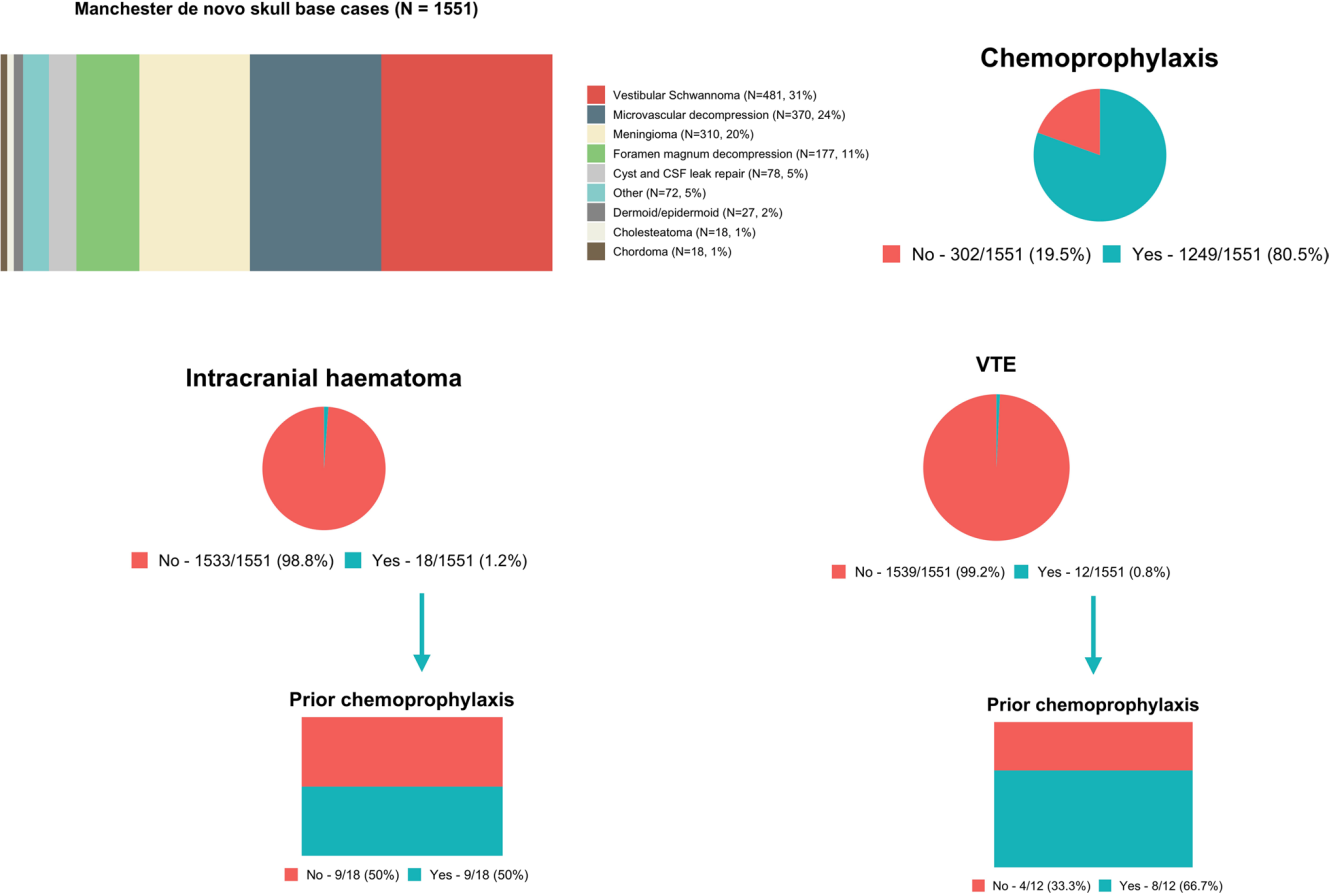
Postoperative risk of intracranial haematoma and venous thromboembolism after skull base surgery. This figure summarises the study results overall. In a cohort of 1551 patients undergoing first-time skull base surgery, the overall risk of haematoma formation was 1.2% and the risk of a DVT/PE was 0.8%. Chemoprophylaxis was used in 80.5% of patients and did not negate the risk of VTE events overall

There were 31 postoperative haematomas, of which 18 were intracranial (0.8% incidence; Fig. 1). A summary of patients that sustained postoperative intracranial haematomas is displayed in Supplementary Table 1. The median time from operation date to diagnosis of an intracranial haematoma was 2 days (range 0–11 days). Management was either surgery (10/18, 56%) or conservative (8/18, 44%).

Table 3 displays factors that were predictive of postoperative intracranial haematoma. In multivariate analysis, prior chemoprophylaxis was associated with a significantly reduced incidence of postoperative intracranial haematoma formation (*p* < 0.001), and oncological pathology was associated with a significantly increased incidence (*p* < 0.001). Analysis including other haematoma types is provided in Supplementary Table 2 and affirmed these associations.

**Table 3:** Factors predictive of intracranial haematoma. All intracranial haematomas occurred in oncology cases. Cases that received prior chemoprophylaxis had a significantly reduced rate of intracranial haematoma formation. *Note that prior chemoprophylaxis is a distinct factor versus chemoprophylaxis at any point (as in Table 1). Multivariate analysis revealed that prior chemoprophylaxis and oncological procedure type were associated with intracranial haematoma formation. **Odds ratio of oncological versus other procedure types

	Rate of haematoma formation	Univariate analysis	Multivariate analysis
Age (years)			
≤ 52	7/797 (1%)	Fisher’s exact, *p* = 0.35	Not entered
> 52	11/754 (1%)		
Gender			
Male	8/596 (1%)	Fisher’s exact, *p* = 0.63	Not entered
Female	10/955 (1%)		
Antiplatelets or anticoagulants			
No	18/1439 (1%)	Fisher’s exact, *p* = 0.64	Not entered
Yes	0/112 (0%)		
Thrombocytopenia			
No	17/1511 (1%)	Fisher’s exact, *p* = 0.38	Not entered
Yes	1/40 (2%)		
Procedure type/histology			
Oncology	18/927 (2%)	**Chi-squared, *X* = 38.6, *p* < 0.001**	**OR = 5.7 × 10**^**7**^, *p* < 0.001**
Vestibular schwannoma	6/482 (1%)		
Meningioma	10/310 (3%)		
Dermoid/epidermoid	0/27 (0%)		
Chordoma	0/18 (0%)		
Cholesteatoma	0/18 (0%)		
Other	2/72 (3%)		
Microvascular decompression	0/370 (0%)		
Cyst and CSF-related	0/255 (0%)		
Prior chemoprophylaxis*			
No	9/313 (3%)	**Fisher’s exact, *p* = 0.004**	**OR = 0.17, 95% CI = 0.07–0.45, *p* < 0.001**
Yes	9/1238 (1%)		

The mortality rate of patients with haematomas was 2/31. One of these occurred in a patient that had translabyrinthine removal of a vestibular schwannoma that sustained a superficial skin haematoma that was surgically evacuated, but deteriorated from hydrocephalus that was unrelated. The second patient underwent a retrosigmoid craniotomy for a petrous meningioma and sustained an intracranial haematoma on postoperative day 1, which was surgically evacuated, without having ever received chemoprophylaxis, and died on postoperative day 4.

### Venous thromboembolism

There were 12 VTE events overall (1.2%; Fig. 1) within 6 months of surgery including 3 DVTs and 9 PEs. A summary of patients that sustained a VTE event is provided in Supplementary Table 3 and included a single case that sustained a PE within 24 h of surgery, prior to commencement of chemoprophylaxis. The median time from operation date to diagnosis was 8 days (range 1–201 days). Prior use of chemoprophylaxis did not significantly influence the rate of VTE formation (Fisher’s exact, *p* > 0.99). No VTE event was fatal.

### Chemoprophylaxis risk–benefit analysis

Propensity scoring was used to match 302 patients treated with/without chemoprophylaxis (Supplementary Table 4). This data re-affirmed the relative safety of chemoprophylaxis with equivalent rates of intracranial haematomas (*p* > 0.99) and VTE events (*p* > 0.99).

**Fig. 2:**
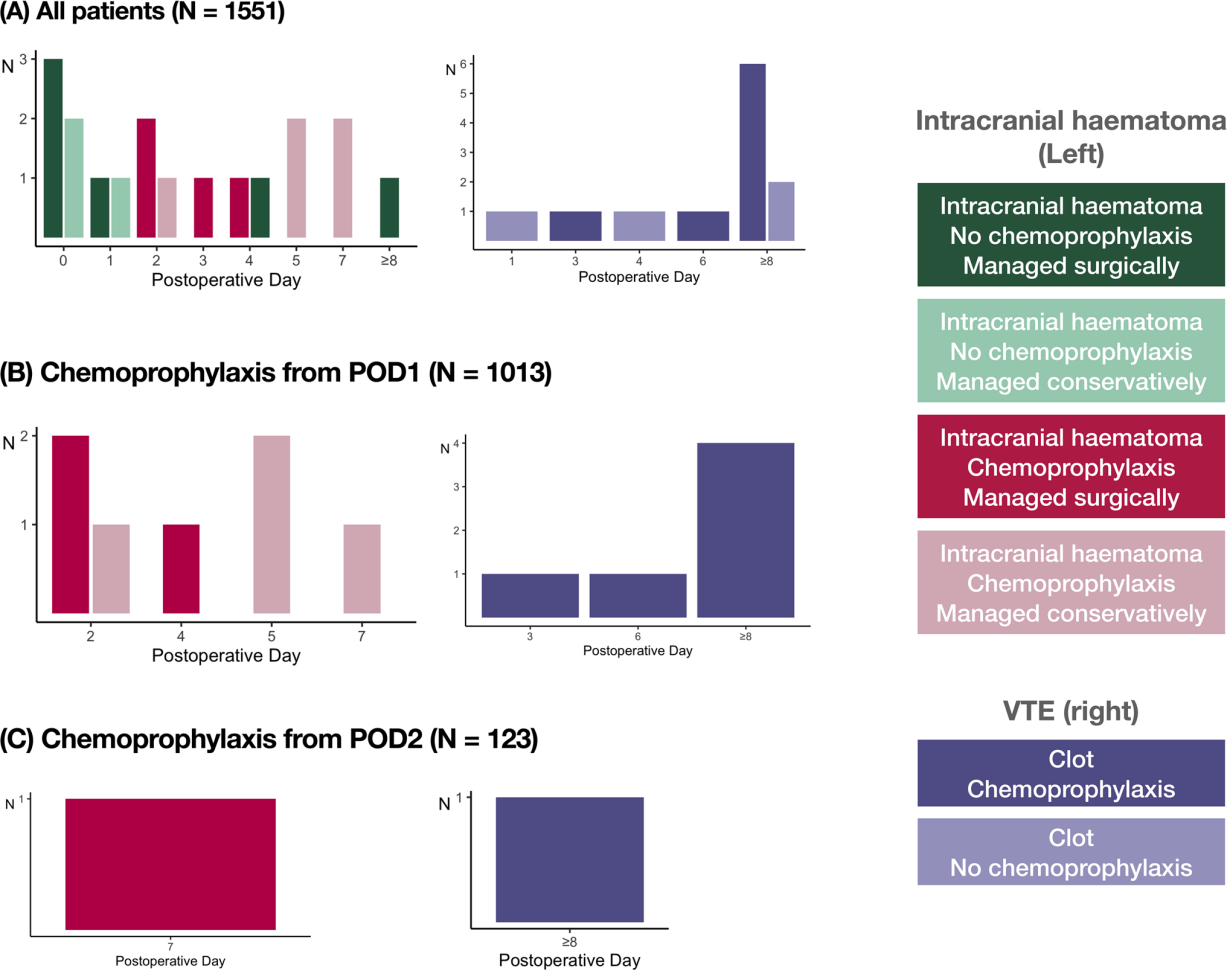
Longitudinal risk of postoperative intracranial haematoma and VTE after skull base surgery. This figure displays the number of intracranial haematomas and VTE events after each successive postoperative day (POD). Data are displayed separately for **A** all patients, **B** patients in whom chemoprophylaxis was commenced on POD1 and **C** patients in whom chemoprophylaxis was commenced on POD2. There was no significant difference in the overall rate of post-chemoprophylaxis intracranial haematomas between patients commenced on chemoprophylaxis on POD1 versus POD2 (Fisher’s exact, *p* = 0.60). This data also demonstrates a relatively low rate of VTE overall that was not negated entirely with chemoprophylaxis

Figure 2 displays the number of intracranial haematomas and VTE events diagnosed on each postoperative day. The use of chemoprophylaxis did not negate the risk of VTE entirely and was highest on/after postoperative day 6 (9/12 VTE events).

The risk of intracranial haematoma formation was particularly high in the first 48 h after surgery. Then, 10/18 (56%) intracranial haematomas occurred within 48 h of surgery, of which 6/10 required surgical intervention (Supplementary Table 1). This is compared to 4/8 patients requiring surgery for intracranial haematomas occurring beyond 48 h (Supplementary Table 1).

### Chemoprophylaxis on postoperative day 1 versus 2

The overall rate of post-chemoprophylaxis intracranial haematoma formation was equivalent between patients administered chemoprophylaxis from postoperative day 1 (7/1013, 0.7%) and postoperative day 2 (1/123, 0.8%; Fisher’s exact, *p* = 0.60). The overall rate of post-chemoprophylaxis VTE was also similar for these groups, respectively (6/1013, [0.6%] vs. 1/123 [0.8%], Fisher’s exact, *p* = 0.60).

Propensity scoring was used to match 123 patients treated with chemoprophylaxis on postoperative days 1 and 2 (Supplementary Table 5). The rate of post-chemoprophylaxis intracranial haematomas was not significantly different between postoperative day 1 (0/123) versus day 2 (1/123, 1%; Fisher’s exact, *p* > 0.99). The rates of VTE were also comparable between postoperative day 1 (1/123, 1%) versus day 2 (1/123, 1%; Fisher’s exact, *p* > 0.99).

## Discussion

In this novel study, we presented data on haematoma and VTE risk after skull base surgery. In a large cohort of 1551 patients, of which over half had oncological pathology, chemoprophylaxis was used in 80% of patients usually from the first postoperative day. The overall risks of postoperative intracranial haematoma and VTE were 1.2% and 0.8%, respectively. The overall rate of VTE was relatively low and not negated entirely even with chemoprophylaxis. Use of chemoprophylaxis was safe and associated with a reduced overall risk of postoperative intracranial haematoma formation, which was highest in the first 48 h of surgery. We further presented data on the risks/benefits of chemoprophylaxis on different postoperative days and found equivalent outcomes when chemoprophylaxis was commenced on postoperative day 1 versus 2.

The proportion of patients receiving chemoprophylaxis in the present study was much greater than other large neuro-oncological series describing rates of 30–40% that have also found a tendency for a lower rate of chemoprophylaxis with primary brain tumours [26]. The widespread reluctance to use chemoprophylaxis routinely in patients undergoing intracranial surgery is likely due to the perceived risks of postoperative intracranial haematomas, which can be associated with significant morbidity, especially in skull base patients. Indeed, in the traditional sense, the possibility of a potentially fatal PE (0% incidence in the present series) has to be weighed up against the possibility of a fatal/life-changing haematoma (< 1% incidence). In some centres, postoperative imaging is routinely employed to guide decision-making relating to postoperative chemoprophylaxis. However, our data demonstrates the relative safety of chemoprophylaxis from the first postoperative day based on clinical grounds without the use of routine imaging. Indeed, patients in whom it was utilised had a reduced incidence of postoperative intracranial haematomas. In a retrospective analysis, this finding could be attributed to use in patients with a lower perceived risk of developing a haematoma, so we place greater emphasis on our propensity-score matched outcomes.

**Fig. 3:**
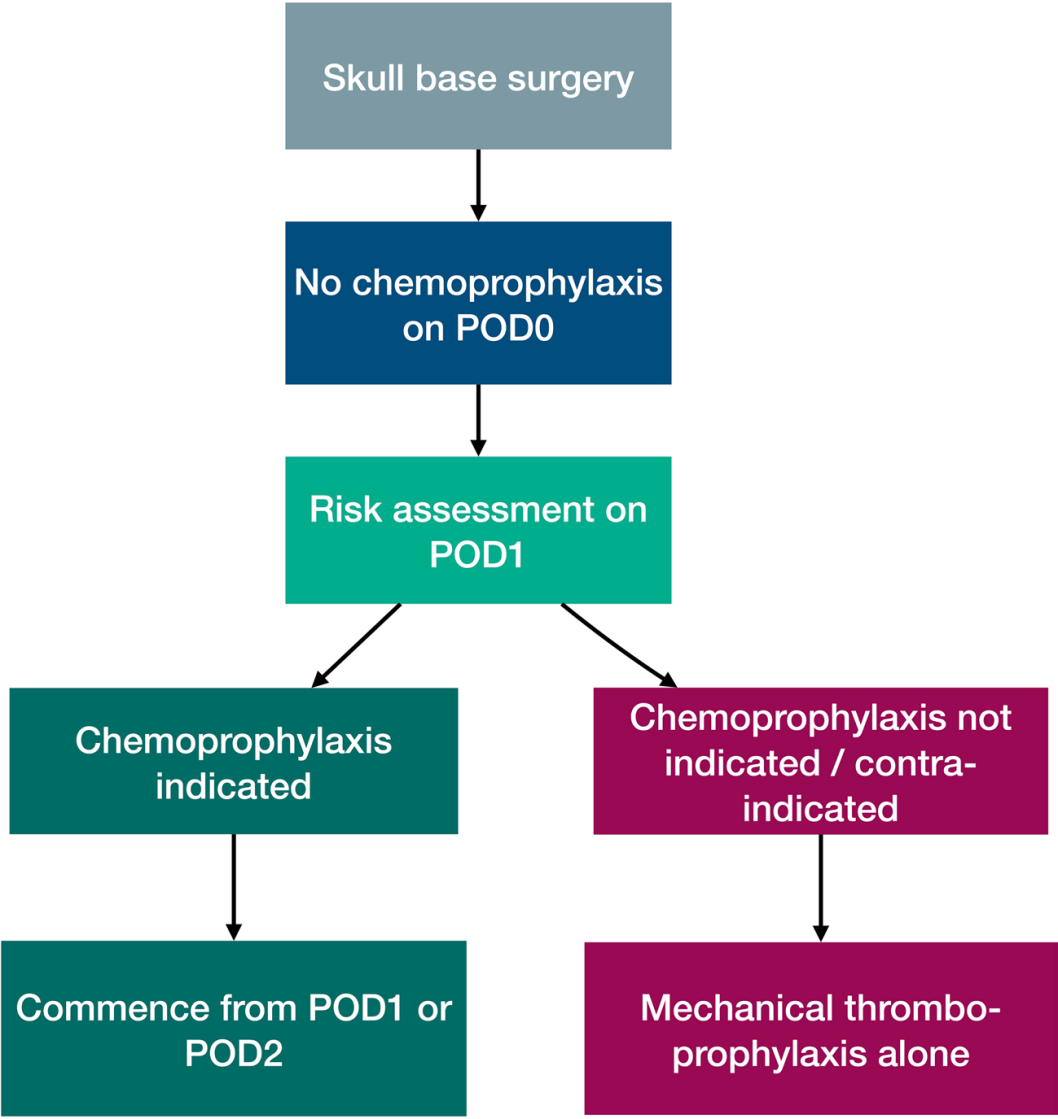
Chemoprophylaxis after skull base surgery. Skull base procedures are associated with an elevated but relatively low risk of VTE, which is apparent from postoperative day 1 and not negated entirely even with chemoprophylaxis. Chemoprophylaxis represents a safe strategy to utilise in this patient group. Chemoprophylaxis is discouraged on the day of surgery given the elevated early risk of spontaneous intracranial haematomas. An individualised risk assessment should occur on postoperative day 1. If chemoprophylaxis is indicated, it can be commenced on postoperative day 1 or 2, which have comparable safety and efficacy profiles. If chemoprophylaxis is not indicated or contraindicated, patients should be offered mechanical thromboprophylaxis measures alone, which still result in a relatively low rate of VTE. We do not advocate post-discharge chemoprophylaxis. Abbreviations: POD, postoperative day

There was a relatively low rate of intracranial haematomas in the present series despite the relatively high utilisation of chemoprophylaxis. Other studies have reported a higher rate of haematomas ranging from 2 to 4%, albeit with different intracranial pathologies [13]. Mortality that was potentially attributable to an intracranial haematoma occurred only in a single case, and there was no mortality attributable to an intracranial haematoma in a patient that received chemoprophylaxis. Overall, therefore, our data demonstrates that, in patients who make it to their first postoperative day without bleeding-related complication, chemoprophylaxis is safe but can also be deferred to the second postoperative day. This is because we observed the highest incidence of intracranial haematomas within the first 48 h of surgery. In patients in whom chemoprophylaxis is contraindicated or deemed too risky, the risk of VTE remains low with mechanical measures alone. We only found oncological pathology as a baseline variable that increased the risk of an intracranial haematoma, but other intraoperative factors (e.g. haemostasis/procedural time and surgical adjuncts) could also be contributory given the timing of most haematomas and should be explored in future [22]. Our data support a personalised approach to chemoprophylaxis in patients undergoing skull base surgery (Fig. 3). This is a useful resource given the lack of dedicated postoperative VTE prophylaxis protocols in different neurosurgical pathologies [13]. Developing such tools is useful given the strong rationale for tailoring postoperative VTE prophylaxis in surgical patients [18].

The rate of VTE in this series was relatively low, although not negligible given the occurrence of VTE events prior to commencement of chemoprophylaxis and the occurrence of VTE events whilst on chemoprophylaxis. This is likely multifactorial and could be attributed to the significant duration of skull base procedures, the high incidence of oncological pathology and requirement for postoperative high-dependency care that itself can involve prolonged immobility. Our data therefore supports prior studies reporting an elevated VTE risk after prolonged (> 45 min) surgery for oncological pathology [8]. Our reported rates of VTE are also in keeping with prior meta-analyses describing reduction in VTE after chemoprophylaxis [14, 24]. This also corroborates our experience with trans-sphenoidal pituitary surgery where a similarly high rate of chemoprophylaxis (73%) yielded zero VTE events in 651 patients within 3 months of surgery [25]. Notably, our VTE rate was also lower than a prior series that utilised delayed postoperative chemoprophylaxis after 72 h in skull base patients, reporting a VTE rate of 10% through serial venous Doppler ultrasound scans [2]. We also only reported data on acquired VTE as defined by DVT/PE, not including cerebral venous sinus thrombosis, which is attributed to surgery near dural venous sinuses and therefore has a high incidence in this patient group [1, 9].

We do not employ post-discharge chemoprophylaxis for skull base patients. This practise is utilised in some patient groups postoperatively for a limited time period when the rates of VTE are particularly high/patient mobility is affected by surgery [7]. Our data does not support this practise for skull base surgery as at least 25% of VTE events occurred beyond 4 weeks—which may be considered a reasonable time period to offer post-discharge chemoprophylaxis (Supplementary Table 3). Furthermore, our patient demographic tends to be fully mobile in the weeks following surgery.

Limitations of the present study include its single-centre design and retrospective data extraction, albeit with interrogation of prospective databases. Over the study period, there was a change in practice towards increased VTE prophylaxis, and bias from this effect was minimised using propensity score matching. Matched comparisons between patients did not consider intention to treat. Factors that therefore determined the decision to commence chemoprophylaxis at all or on postoperative day 1 versus 2 were not clear. Data were not available on variables that are associated with VTE risk such as diabetes mellitus, body mass index, ethnicity, and smoking history [12, 15].

## Conclusion

In this study which represents the first of its kind, we described the benefits and risks of chemical thromboembolism prophylaxis (chemoprophylaxis) after skull base surgery. Skull-base procedures are associated with a relatively low risk (0.8%) of VTE in our cohort. There is also a low rate of postoperative intracranial haematomas (1.2%), which are most likely to occur in the first 48 h of surgery. Chemoprophylaxis represents a safe strategy to reduce the risk of DVT/PE without significantly increasing the risk of postoperative haematoma. Our data demonstrates similar VTE/intracranial haematoma rates between patients commenced on chemoprophylaxis from postoperative day 1 versus 2. We therefore support a personalised approach to chemoprophylaxis, considering it from postoperative days 1 or 2 when indicated. Future studies should consider the timing of chemoprophylaxis in relation to the VTE/haematoma risk in this patient group.

## Supplementary Information

Below is the link to the electronic supplementary material.

Supplementary file1 (DOCX 54 KB)

## Data Availability

Not applicable.
