# Supplementary material for: Venous thromboembolism chemical prophylaxis after skull base surgery
Source: Acta Neurochir (Wien). 2024 Apr 3;166(1):165. doi: 10.1007/s00701-024-06035-9 (PMC10987339; doi:10.1007/s00701-024-06035-9)
Supplement: Supplementary file 1 — Supplementary file1 (DOCX 54 KB) [file 701_2024_6035_MOESM1_ESM.docx]

**Supplementary Table 1. Clinical characteristics of patients sustaining a postoperative intracranial haematoma**. Cases are sorted by postoperative date of diagnosis.

| **Age** | **Gender** | **Antiplatelets** | **Anticoagulants** | **Diagnosis** | **Prior chemoprophylaxis** | **POD diagnosed** | **Management** | **VTE** | **Survival** |
| --- | --- | --- | --- | --- | --- | --- | --- | --- | --- |
| 58 | Female | No | No | Meningioma | No | 0 | Conservative | None | Alive |
| 30 | Male | No | No | Vestibular Schwannoma | No | 0 | Surgery | None | Alive |
| 47 | Female | No | No | Metastasis | No | 0 | Surgery | None | Alive |
| 62 | Female | No | No | Meningioma | No | 0 | Conservative | None | Alive |
| 50 | Male | No | No | Meningioma | No | 0 | Surgery | None | Alive |
| 67 | Male | No | No | Vestibular Schwannoma | No | 1 | Conservative | None | Alive |
| 69 | Female | No | No | Meningioma | No | 1 | Surgery | None | Death (POD4) |
| 61 | Female | No | No | Vestibular Schwannoma | Tinzaparin | 2 | Surgery | PE | Alive |
| 68 | Female | No | No | Vestibular Schwannoma | Tinzaparin | 2 | Surgery | None | Alive |
| 68 | Male | No | No | Meningioma | Tinzaparin | 2 | Conservative | None | Alive |
| 72 | Female | No | No | Vestibular Schwannoma | Tinzaparin | 3 | Surgery | None | Alive |
| 40 | Female | No | No | Chondrosarcoma | No | 4 | Surgery | None | Alive |
| 55 | Male | No | No | Meningioma | Tinzaparin | 4 | Surgery | None | Alive |
| 34 | Male | No | No | Vestibular Schwannoma | Tinzaparin | 5 | Conservative | None | Alive |
| 63 | Male | No | No | Meningioma | Tinzaparin | 5 | Conservative | None | Alive |
| 40 | Female | No | No | Meningioma | Tinzaparin | 7 | Conservative | None | Alive |
| 37 | Male | No | No | Meningioma | Tinzaparin | 7 | Conservative | None | Alive |
| 53 | Female | No | No | Meningioma | No | 11 | Surgery | None | Alive |

**Supplementary Table 2. Factors predictive of any type of postoperative haematoma.** There were 31 haematomas of any type overall. The median time from operation date to diagnosis was 2 days (range 0-57 days). This figure included three abdominal haematomas requiring surgery diagnosed at 15, 51 and 57 days postoperatively. 15/31 (48%) haematomas occurred in patients that had received at least one dose of chemoprophylaxis. In multivariate logistic regression, oncological procedure type and prior chemoprophylaxis were associated with haematoma formation. *Odds ratio of oncological versus other procedure types.

|  | **Rate of haematoma formation** | **Univariate analysis** | **Multivariate analysis** |
| --- | --- | --- | --- |
| **Age (years)** |  |  |  |
| ≤52 | 12/795 (2%) | Fisher’s Exact, p = 0.20 | Not entered |
| >52 | 19/753 (3%) |  |  |
| **Gender** |  |  |  |
| Male | 16/596 (3%) | Fisher’s Exact, p = 0.14 | OR = 0.61, 95% CI 0.30-1.26, p = 0.19 |
| Female | 15/955 (2%) |  |  |
| **Antiplatelets or anticoagulants** |  |  |  |
| No | 30/1439 (2%) | Fisher’s Exact, p = 0.72 | Not entered |
| Yes | 1/112 (1%) |  |  |
| **Thrombocytopenia** |  |  |  |
| No | 29/1511 (2%) | Fisher’s Exact, p = 0.19 | Not entered |
| Yes | 2/40 (5%) |  |  |
| **Procedure type/histology** |  |  |  |
| **Oncology** | 27/927 (3%) | **Chi-squared, X = 11.7, p = 0.003** | **OR = 5.95, 95% CI 2.04-17.35, p<0.001*** |
| Vestibular Schwannoma | 13/482 (3%) |  |  |
| Meningioma | 10/310 (3%) |  |  |
| Dermoid/epidermoid | 0/27 (0%) |  |  |
| Chordoma | 0/18 (0%) |  |  |
| Cholesteatoma | 1/18 (6%) |  |  |
| Other | 3/72 (4%) |  |  |
| **Microvascular decompression** | 0/370 (0%) |  |  |
| **Cyst and CSF related** | 4/254 (2%) |  |  |
| **Prior chemoprophylaxis** |  |  |  |
| No | 15/313 (5%) | **Fisher’s Exact, p<0.001** | **OR = 0.21, 95% CI 0.10-0.43, p<0.001** |
| Yes | 16/1238 (1%) |  |  |

**Supplementary Table 3. Clinical characteristics of patients sustaining a VTE event.** Cases are sorted by postoperative date of diagnosis. *This patient sustained a within 24 hours of surgery, prior to the conventional start time of chemoprophylaxis. They were classified in the chemoprophylaxis group due to the intention to treat them as such and the fact they received anticoagulation from postoperative day 1 onwards due to the new diagnosis. Abbreviations: POD = postoperative day; AF = atrial fibrillation; CKD = chronic kidney disease; IHD = ischaemic heart disease; PE = pulmonary embolism; DVT = deep vein thrombosis; LMWH = low molecular weight heparin.

| **Age** | **Gender** | **Antiplatelets** | **Anticoagulants** | **Risk factors** | **Diagnosis** | **Chemo-prophylaxis** | **POD diagnosed** | **Type** | **Treatment** |
| --- | --- | --- | --- | --- | --- | --- | --- | --- | --- |
| 60 | Female | No | Warfarin | IHD | Vestibular Schwannoma | None* | 1 | PE | Warfarin |
| 55 | Male | No | No | Nil | Microvascular decompression | Tinzaparin | 3 | PE | Warfarin |
| 57 | Female | No | No | Nil | Other | None | 4 | PE | Warfarin |
| 61 | Female | No | No | Nil | Vestibular Schwannoma | Tinzaparin | 6 | PE | None |
| 59 | Female | No | No | Obesity | Vestibular Schwannoma | Tinzaparin | 8 | PE | Rivaroxaban |
| 54 | Male | No | No | Nil | Vestibular Schwannoma | None | 8 | PE | Warfarin |
| 38 | Female | No | No | Obesity | Meningioma | Tinzaparin | 20 | DVT | LMWH |
| 63 | Female | No | No | Nil | Microvascular decompression | Tinzaparin | 24 | PE | Rivaroxaban |
| 50 | Female | No | No | Nil | Meningioma | Tinzaparin | 35 | PE | LMWH |
| 42 | Female | No | No | Obesity | Meningioma | Tinzaparin | 35 | DVT | LMWH |
| 80 | Female | No | No | Nil | Meningioma | Enoxaparin | 116 | PE | LMWH |
| 78 | Male | No | Warfarin | CKD, AF | Meningioma | Tinzaparin | 201 | DVT | Warfarin |

**Supplementary Table 4. Propensity score matched comparison of patients that received chemoprophylaxis from postoperative day 1 versus those that did not receive any chemoprophylaxis**. *Postoperative day 2 onwards to allow a fair comparison. This table demonstrates equivalent intracranial haematoma/VTE outcomes between these groups, indicating the safety of chemoprophylaxis.

|  | **Chemoprophylaxis from postoperative day 1**  **(N = 302)** | **No chemoprophylaxis**  **(N = 302)** | **Comparison** |
| --- | --- | --- | --- |
| **Age (years)** |  |  |  |
| Median | 53 | 53 | t-test, t = 0.05, p = 0.96 |
| Range | 17-62 | 16-62 |  |
| **Gender** |  |  |  |
| Male | 120 (40%) | 122 (40%) | Fisher’s Exact, p = 0.93 |
| Female | 182 (60%) | 180 (60%) |  |
| **Pre-op antiplatelets or anticoagulants** |  |  |  |
| No | 288 | 288 | Fisher’s Exact, p>0.99 |
| Yes | 14 | 14 |  |
| **Surgical approach** |  |  |  |
| Translabyrinthine/transmastoid | 97 (32%) | 97 (32%) | Chi-squared, χ2 = 0.20, p>0.99 |
| Retrosigmoid | 112 (37%) | 113 (37%) |  |
| Foramen magnum decompression | 50 (17%) | 49 (16%) |  |
| Frontal/pterional | 12 (4%) | 11 (4%) |  |
| Endonasal | 10 (3%) | 9 (3%) |  |
| Other | 21 (7%) | 23 (8%) |  |
| **Type of procedure/histology** |  |  |  |
| Oncology | 134 (44%) | 133 (44%) | Chi-squared, χ2 = 0.01, p>0.99 |
| Microvascular decompression | 99 (33%) | 99 (33%) |  |
| Cyst and CSF related | 69 (23%) | 70 (23%) |  |
| **Intracranial haematomas*** | 2 (1%) | 2 (1%) | Fisher’s Exact, p>0.99 |
| **Venous thromboembolism** | 1 (<1%) | 2 (1%) | Fisher’s Exact, p>0.99 |

**Supplementary Table 5. Propensity score matched comparison of patients that received chemoprophylaxis from postoperative day 1 versus 2**. This table demonstrates equivalent intracranial haematoma/VTE outcomes between these groups, including no significant difference in post-chemoprophylaxis haematomas or VTE events.

|  | **Postoperative day 1**  **(N = 123)** | **Postoperative day 2**  **(N = 123)** | **Comparison** |
| --- | --- | --- | --- |
| **Age (years)** |  |  |  |
| Median | 50 | 50 | t-test, t = 0.12, p = 0.91 |
| Range | 18-81 | 18-81 |  |
| **Gender** |  |  |  |
| Male | 44 (36%) | 44 (36%) | Fisher’s Exact, p>0.99 |
| Female | 79 (64%) | 79 (64%) |  |
| **Pre-op antiplatelets or anticoagulants** |  |  |  |
| No | 116 (94%) | 116 (94%) | Fisher’s Exact, p>0.99 |
| Yes | 7 (6%) | 7 (6%) |  |
| **Surgical approach** |  |  |  |
| Translabyrinthine/transmastoid | 45 (37%) | 45 (37%) | Chi-squared, χ2 = 0.18, p>0.99 |
| Retrosigmoid | 31 (25%) | 31 (25%) |  |
| Foramen magnum decompression | 10 (8%) | 10 (8%) |  |
| Frontal/pterional | 17 (14%) | 17 (14%) |  |
| Endonasal | 8 (7%) | 7 (6%) |  |
| Other | 12 (10%) | 13 (11%) |  |
| **Type of procedure/histology** |  |  |  |
| Oncology | 82 (67%) | 82 (67%) | Chi-squared, χ2 <0.01, p>0.99 |
| Microvascular decompression | 20 (16%) | 20 (16%) |  |
| Cyst and CSF related | 21 (17%) | 21 (17%) |  |
| **Post-chemoprophylaxis intracranial haematoma** | 0 (0%) | 1 (1%) | Fisher’s Exact, p>0.99 |
| **Venous thromboembolism** | 1 (1%) | 1 (1%) | Fisher’s Exact, p>0.99 |
